# Supplementary figures and images for: Ultrasensitive quantification of serum IFN-α and IFN-γ in systemic lupus erythematosus: A cross-sectional observational study
Source: PLoS Med. 2025 Dec 5;22(12):e1004841. doi: 10.1371/journal.pmed.1004841 (PMC12680241; doi:10.1371/journal.pmed.1004841)

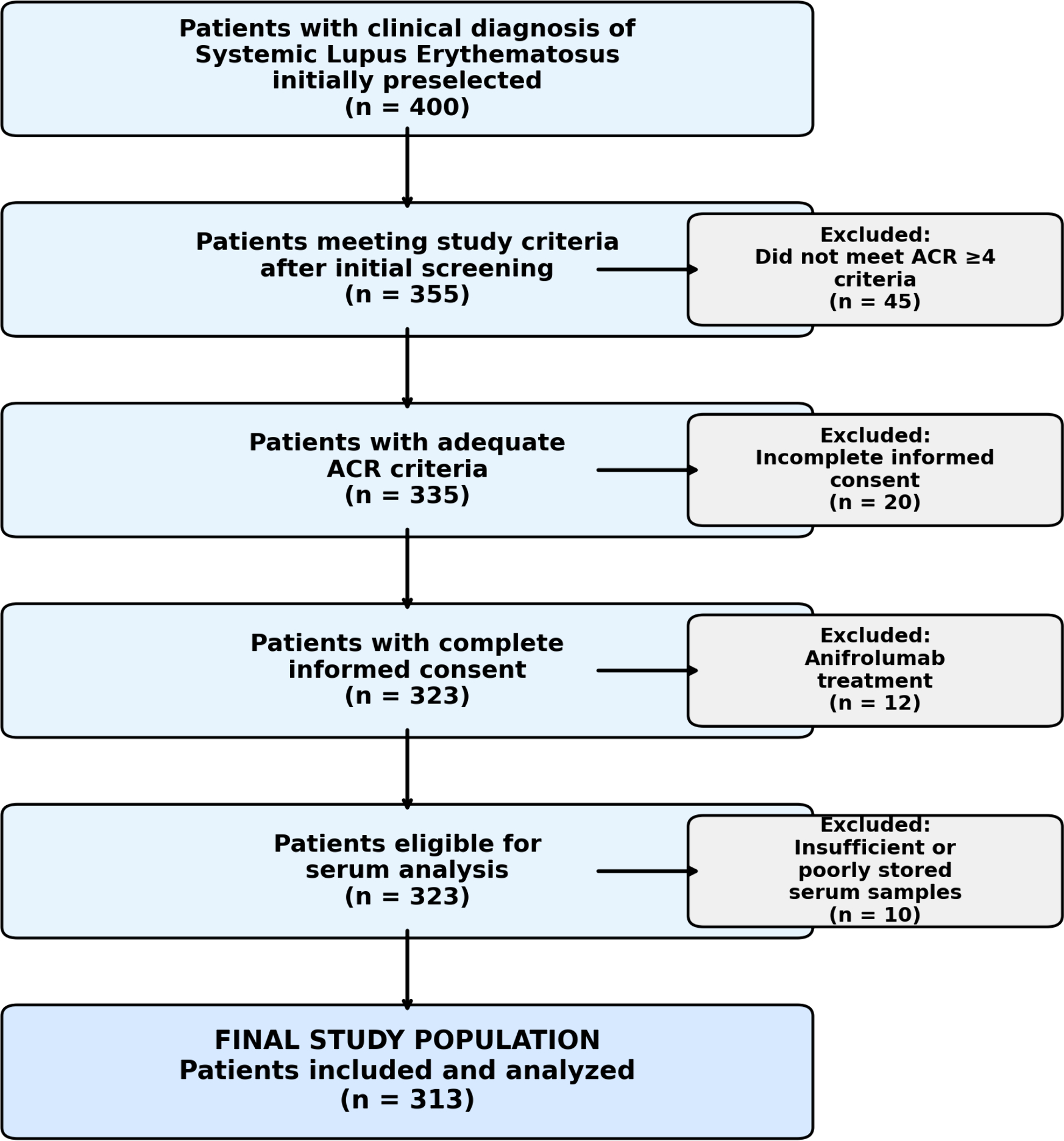

Supplement: S1 Fig — Patient selection flowchart showing inclusion of 313 patients from 400 initially screened individuals with systemic lupus erythematosus. (TIF) [file pmed.1004841.s001.tif]

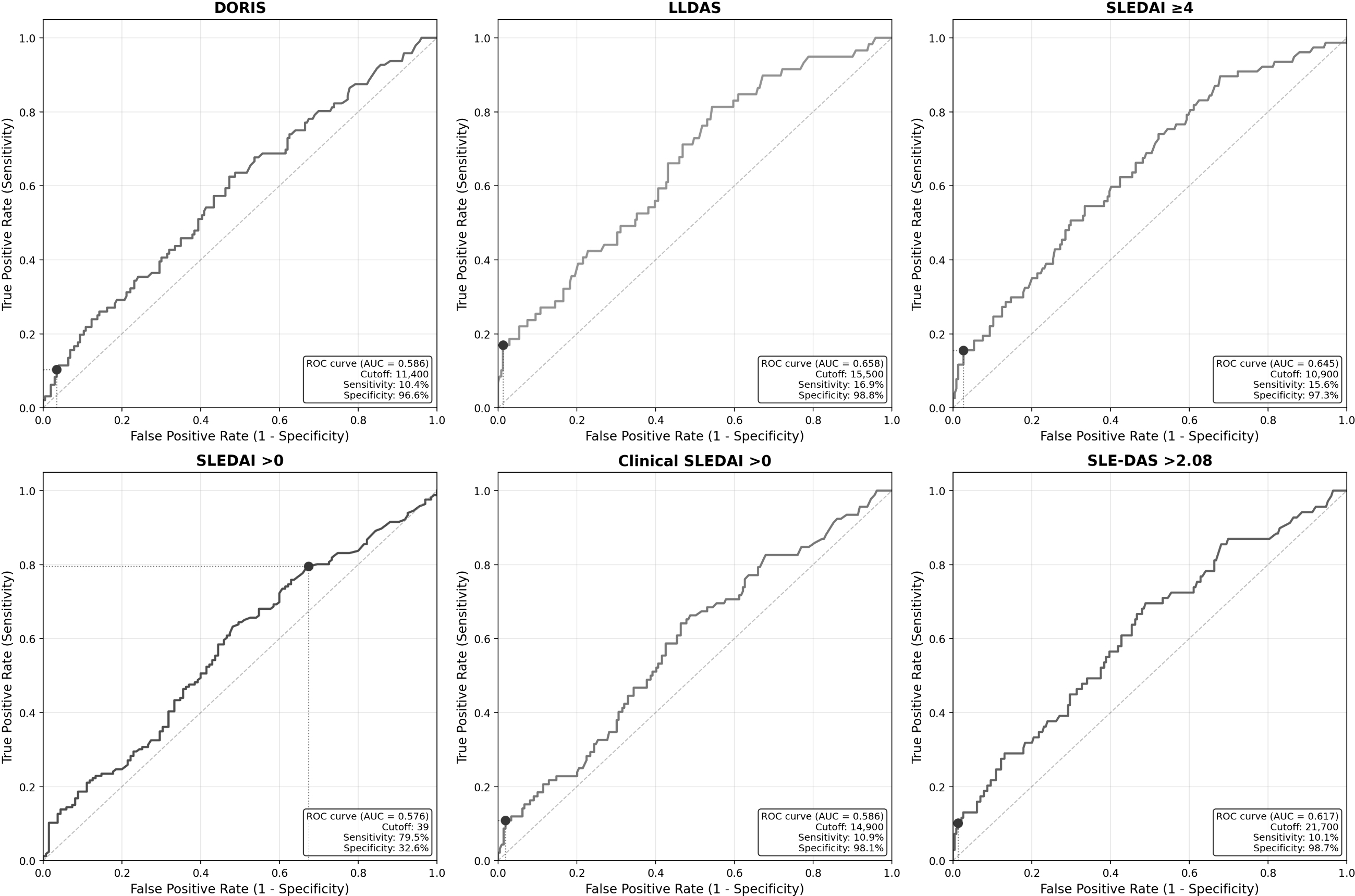

Supplement: S2 Fig — SLEDAI, Systemic Lupus Erythematosus Disease Activity Index; SLE-DAS, SLE Disease Activity Score; DORIS, Definitions of Remission in SLE; LLDAS, Lupus Low Disease Activity State; Clinical SLEDAI-2k omits complement and anti-dsDNA components from original SLEDAI-2K. (TIF) [file pmed.1004841.s002.tif]
