# Supplementary material for: Ultrasensitive quantification of serum IFN-α and IFN-γ in systemic lupus erythematosus: A cross-sectional observational study
Source: PLoS Med. 2025 Dec 5;22(12):e1004841. doi: 10.1371/journal.pmed.1004841 (PMC12680241; doi:10.1371/journal.pmed.1004841)
Supplement: S1 Table — (DOCX) [file pmed.1004841.s003.docx]

| **S1 Table. Diagnostic performance (area under the curve) of IFN-α levels to distinguish disease activity as defined by several activity ~~damage~~ scores.** | | | | | | | | | | | | | | | | | | | | | | | | | | | | | | | | | |  | |  |  |
| --- | --- | --- | --- | --- | --- | --- | --- | --- | --- | --- | --- | --- | --- | --- | --- | --- | --- | --- | --- | --- | --- | --- | --- | --- | --- | --- | --- | --- | --- | --- | --- | --- | --- | --- | --- | --- | --- |
|  |  | N | | | | AUC [95% CI] | | | Optimal cutoff, femtograms/ml | | |  | Sensitivity  [95% CI], % | | | | |  | | Specificity  [95% CI], % | | | | PPV, % | | | NPV, % | | | | | |  |  |  |  |  |
| Non-DORIS | | 99/212 | | | | 0.586 [0.517, 0.656] | | | | 11,400 | |  | | 10 [7, 19] | | |  | | 96 [92, 98] | | | | 58 | | | 70 | | | | | |  |  |  |  |  |  |
| Non-LLDAS | | 63/249 | | | | 0.658 [0.582, 0.734] | | | | 15,500 | |  | | 17 [9, 29] | | |  | | 99 [96, 99] | | | | 77 | | | 83 | | | | | |  |  |  |  |  |  |
| Non-LLDAS [Modified] | | | | 64/248 | | 0.663 [0.589, 0.739] | | | | 15,500 | |  | | 17 [9, 28] | | |  | | 99 [96, 99] | | | | 77 | | | 83 | | | | | |  |  |  |  |  |  |
| SLEDAI-2k | |  | | | |  | | | |  | |  | |  | | |  | |  | | | |  | | |  | | | | | |  |  |  |  |  |  |
|  | >=4 | 80/233 | | | | 0.645 [0.574, 0.715] | | | | 10,900 | |  | | 16 [9, 25] | | |  | | 97 [94, 99] | | | | 67 | | | 77 | | | | | |  |  |  |  |  |  |
| >0 | | | 172/141 | | | | 0.576 [0.511, 0.641] | | | 39 | |  | | 80 [73, 85] | | |  | | 33 [25, 41] | | | | 59 | | | | | 56 | |  |  |  |  |  |  |  |  |
| Clinical SLEDAI2K | | | | |  | | |  | | |  | | | |  |  | | | | |  |  | | |  | | | |  | |  |  |  |  |  |  |  |
| >0 | | 96/217 | | | | 0.587 [0.517, 0.656] | | | | 14,900 | |  | | 11 [6, 19] | | |  | | 98 [95, 99] | | | | 71 | | | 71 | | | | | |  |  |  |  |  |  |
| SLEDAS | |  | | | |  | | | |  | |  | |  | | |  | |  | | | |  | | |  | | | | | |  |  |  |  |  |  |
|  | >2.08 | 72/237 | | | | 0.617 [0.540, 0.693] | | | | 21,700 | |  | | 10 [3, 16] | | |  | | 99 [97, 99] | | | | 83 | | | 78 | | | | | |  |  |  |  |  |  |
| SLEDAI-2K: Systemic Lupus Erythematosus Disease Activity Index; SLE-DAS: SLE Disease Activity Score; DORIS: Definitions of Remission in SLE; LLDAS: Lupus Low Disease Activity State; Modified LLDAS uses ≤5 mg/day prednisone criteria instead of ≤7.5 mg; Clinical SLEDAI-2k omits complement and anti-dsDNA components from original SLEDAI-2K. AUC: area under the curve; PPV: positive predictive value; NPV: negative predictive value. | | | | | | | | | | | | | | | | | | | | | | | | | | | | | | | | | | |  |  |  |
