# Supplementary material for: Ultrasensitive quantification of serum IFN-α and IFN-γ in systemic lupus erythematosus: A cross-sectional observational study
Source: PLoS Med. 2025 Dec 5;22(12):e1004841. doi: 10.1371/journal.pmed.1004841 (PMC12680241; doi:10.1371/journal.pmed.1004841)
Supplement: S1 Protocol — (DOCX) [file pmed.1004841.s005.docx]

**Ultrasensitive Quantification of Serum IFN-α, But Not IFN-γ, Reflects Inflammation, Disease Activity, and Autoantibody Status in Systemic Lupus Erythematosus**

**Study protocol**

1. Study Type.

1.1. Single-center, three-year cross-sectional study in patients with rheumatoid arthritis (RA), systemic lupus erythematosus (SLE), and controls to estimate the relationships between serum expression and gene signature of type I, II, and III interferons (and subtypes) and carotid intima-media thickness, plaque presence, and carotid stiffness in patients with RA, SLE, and controls. Additionally, this study will assess the relationship between alterations in serum and gene expression with other cardiovascular disease determinants such as lipid profile, insulin resistance, beta cell dysfunction, reverse cholesterol transport, etc.

1.2. A six-month prospective study in a subgroup of RA patients initiating anti-TNF therapies. Eligible patients starting anti-TNF therapy will be studied before and after six months of treatment. We will determine if the modifications in IFN molecule levels and expression change after treatment and if changes also occur in lipid profile and insulin resistance associated with potential changes in IFN. Ultrasound techniques will not be performed during this second visit on these patients.

1.3. Variables.

1.3.1. Primary outcome variable (dependent): carotid artery intima-media thickness and plaque presence, carotid stiffness measurement (endothelial dysfunction); reverse cholesterol transport; insulin resistance and beta cell dysfunction.

1.3.2. Secondary variables: explanatory (independent) variables: serum concentrations and gene expression of IFN types I, II, and III and subtypes. Other dependent variables: a) abnormal lipid profile including paraoxonase/arylesterase and myeloperoxidase antioxidant activity, cholesterol ester transfer protein activity, lecithin-cholesterol acyltransferase, reverse cholesterol transport measurements, PCSK9 (Proprotein Convertase Subtilisin/Kexin Type 9) levels; b) insulin resistance indices and beta cell function (measured via serum insulin and C-peptide and calculated using HOMA2 – homeostatic model assessment). Confounding variables: sex, age, body mass index, obesity, blood pressure, antihypertensive treatment, fasting glucose, presence of diabetes, smoking, statin use.

Demographic data, surgical history, smoking, vascular comorbidities, and current treatment will be recorded. Weight and height will be measured to determine body mass index (BMI) (body weight divided by height squared in kg/m2) and waist-to-hip ratio. Disease activity will be assessed using DAS28 (Disease Activity Score 28 Joints), CDAI (Clinical Disease Activity Index), or SDAI (Simple Disease Activity Index), and HAQ (Health Assessment Questionnaire). Metabolic syndrome will be defined following the 2005 National Cholesterol Education Program Adult Treatment Panel III (ATP III) criteria. Cardiovascular risk in the Spanish population will be assessed via the SCORE2 (Systematic Coronary Risk Evaluation version 2 updated). Disease activity and damage in SLE will be evaluated through the Systemic Lupus Erythematosus Disease Activity Index (SLEDAI-2K) and the SLICC/ACR damage index (SDI), respectively. Disease severity will also be assessed by the Katz index.

2. Population Selection

2.1. Inclusion Criteria: 1. Men or women not pregnant or breastfeeding; 2. ≥18 years and <70 years old; 3. Patients under any disease-modifying drug treatment (including those already receiving biologicals) or initiating biological treatment (anti-TNF or others); 4. If receiving oral corticosteroids, dose must be ≤ 10 mg prednisone and stable for at least one month prior to inclusion; 5. Patients capable and willing to sign informed consent.

2.2. Exclusion Criteria: Patients with any of the following will be ineligible: 1. Autoimmune rheumatic diseases other than RA or SLE, such as mixed connective tissue disease, scleroderma, polymyositis, or systemic involvement secondary to RA (e.g., vasculitis, pulmonary fibrosis, or Felty’s syndrome). Sjögren’s syndrome with RA or SLE is not an exclusion criterion; 2. Functional Class IV RA as defined by the ACR Functional Status Classification (complete or substantial disability confining the patient to bed or wheelchair with inability for self-care). 3. History or current presence of inflammatory joint disease other than RA (e.g., gout, reactive arthritis, psoriatic arthritis, seronegative spondyloarthropathy, Lyme disease). 4. Glomerular filtration rate under 60 ml/min/1.73 m2 and presence of active renal disease. 5. Pregnant or lactating women. 6. Evidence of uncontrolled severe concomitant cardiovascular, nervous system, pulmonary (including COPD), renal, hepatic, endocrine (including diabetes mellitus), or gastrointestinal diseases, or any which, in the investigator’s judgment, would alter the interferon system profile. 7. Body weight >150 kg or BMI >50 kg/m2. 8. History of alcoholism, drug addiction or substance abuse within six months prior to screening visit.

3.. Data Collection

3.1. Baseline. Data for each patient will be recorded in a Case Report Form (CRF) in paper and electronic formats. CRF data must be verifiable against source documents such as clinical histories, laboratory data, and ultrasound reports. Concentrations obtained by the different assays will be attached to each patient’s CRF with standard curves and validation criteria. The data will be included in an electronic database facilitating statistical analysis. Blood samples will be collected fasting from the antecubital vein during patient and control study visits.

3.2. Carotid Intima-Media Thickness and Plaque Evaluation. Carotid ultrasound will evaluate the thickness of the carotid intima-media (cIMT) in the common carotid artery and detect focal plaques within the extracranial carotid tree in patients and controls. A commercially available ultrasound system, Mylab 70, Esaote (Genoa, Italy), equipped with a 7-12 MHz linear transducer and automated radiofrequency-guided software–Quality Intima Media Thickness Real-Time (QIMT, Esaote, Maastricht, Netherlands)–will be used. Plaque criteria in accessible extracranial carotid tree segments (common carotid artery, bulb, and internal carotid artery) will be based on Mannheim consensus.

3.3. Arterial Stiffness Evaluation. Arterial stiffness will be assessed by ultrasound exam using Mylab 70 (Esaote) equipped with an automatic Quality Arterial Stiffness (QAS) radiofrequency (RF) package and a LA523 vascular probe of 4-13 MHz with RF emission-reception signals. QAS automatically evaluates changes in arterial internal diameter between systolic and diastolic phases, obtaining arterial distension waveform via real-time automatic RF signal analysis. This enables calculation of arterial stiffness and arterial wall stress (pulse wave velocity, PWV in m/s, and augmentation index, AIX)–carotid arterial stiffness index.

3.4. Follow-up Data Collection. A subgroup of patients requiring anti-TNF treatment will be recruited at our Day Hospital for inclusion. Initial visits for these patients will be similar to the case-control study visit schedules. Patients continuing anti-TNF treatment at 6 months will undergo repeated analytical parameter determination.

4. Immunoassays for IFN-induced soluble proteins and IFN-stimulated gene assays. Laboratory techniques for IFN analysis are detailed in the Annex of this submission.

5. Data Analysis Objectives. IFN gene expression scores are typically categorized (low, medium, high), but we will explore continuous and categorical IFN score analysis. Initially, multivariable analyses will compare IFN expression between patients with RA and SLE and controls. The relationship between IFN expression and carotid plaque presence, intima-media thickness, or arterial stiffness will be evaluated using multivariable linear or logistic regression. Differences in IFN effects on atheromatosis or carotid stiffness between diseases or between groups and controls will be assessed by adding interaction terms to regression models to determine if relationships differ among the three populations (RA, SLE, controls). A predictive model will be developed for interferon system molecules that, parsimoniously combined, predict carotid plaque presence or endothelial dysfunction. The relationship between IFN and other cardiovascular risk factors will be studied by multivariable linear/logistic regression. Stata 17 will be used for analysis; statistical significance is defined as a two-sided p-value <0.05.

6. Ethics. The project will be submitted to the Ethics Committee of our center. No patient or control will experience any change in care from the Rheumatology Department. Patients and controls will sign informed consent and will receive a study information sheet outlining objectives and interventions. The processing, communication, and transfer of all participants’ personal data are detailed in this application.

7. Study Limitations, Data Biases, and Compensation Mechanisms. Execution risks and contingency plans will be highlighted. A primary limitation may be the high patient recruitment needed. However, based on prior ISCIII projects, we believe capable of evaluating this patient volume (see previous publications on CV). Collaboration with another regional hospital will facilitate recruitment. Secondly, IFN expression determinations involve different techniques and procedures with particular laboratory requirements. Hence, a clinical biochemistry specialist and an immunologist familiar with gene expression studies will be included in the study. As no clear consensus exists on the most appropriate IFN measurement technique, prior literature was used to select immunoassays for IFN-induced soluble proteins and IFN-stimulated genes (signature). If serum IFN level determinations prove difficult, SiMOA techniques will be employed (see Annex). Statistical analysis of interferon signature is demanding, requiring principal component analysis techniques (see Annex).

8. Novelty, Originality, Relevance, and Institutional Incentive Effect. To our knowledge, no previous studies exist on IFN’s relationship with cardiovascular risk in SLE and RA with the aims of this study. We consider this approach novel and highly relevant given IFN’s emerging role and related new therapies in immune-mediated diseases and cardiovascular risk.

9. Gender Perspective. As evident in CVs and publications, female co-authors generally outnumber males. Co-investigators in this submission are equally balanced by gender. Both RA and SLE predominantly affect women, with female-to-male ratios of approximately 3:4 for RA and 9:1 for SLE, respectively.
