# Supplementary material for: Ultrasensitive quantification of serum IFN-α and IFN-γ in systemic lupus erythematosus: A cross-sectional observational study
Source: PLoS Med. 2025 Dec 5;22(12):e1004841. doi: 10.1371/journal.pmed.1004841 (PMC12680241; doi:10.1371/journal.pmed.1004841)
Supplement: S1 Text — (DOCX) [file pmed.1004841.s006.docx]

**Ultrasensitive Quantification of Serum IFN-α, But Not IFN-γ, Reflects Inflammation, Disease Activity, and Autoantibody Status in Systemic Lupus Erythematosus**

**S1 Text - Abbreviations**

AUC: Area Under the Curve; ANA: Antinuclear Antibodies; CRP: C-reactive Protein; DORIS: Definitions of Remission in SLE; DMARDs: Disease-Modifying Antirheumatic Drugs; DNA: Deoxyribonucleic Acid; ENA: Extractable Nuclear Antigen; ELISA: Enzyme-Linked Immunosorbent Assay; hs-CRP: High Sensitivity C-reactive Protein; IFN: Interferon; IFN-α: Interferon-alpha; IFN-γ: Interferon-gamma; IL: Interleukin; LLDAS: Lupus Low Disease Activity State; PGA: Physician Global Assessment; PPV: positive predictive value; NPV: negative predictive value; ROC: Receiver Operating Characteristic; SDI: Damage Index; SLE: Systemic Lupus Erythematosus; SLEDAI-2K: Systemic Lupus Erythematosus Disease Activity Index 2000; SLE-DAS: SLE Disease Activity Score; SLICC: Systemic Lupus International Collaborating Clinics; Simoa: Single Molecule Array.
