# Supplementary material for: Ultrasensitive quantification of serum IFN-α and IFN-γ in systemic lupus erythematosus: A cross-sectional observational study
Source: PLoS Med. 2025 Dec 5;22(12):e1004841. doi: 10.1371/journal.pmed.1004841 (PMC12680241; doi:10.1371/journal.pmed.1004841)
Supplement: Attachment — Submitted filename: Gonzalez-Gay.docx [file pmed.1004841.s008.docx]

Gonzalez-Gay et al. present a very nice data set relating serum interferon-alpha and -gamma levels measured by high sensitivity Simoa assay to a number of demographic and clinical parameters in 313 patients with SLE. While the observations presented are not novel and generally confirm previously published studies, the availability of IFN levels across a large number of patients allows for a multivariate analysis of correlations that emphasize the significance of IFN-a (but not IFN-gamma) as a mediator with potential to assist in characterizing disease activity and possibly organ damage in the context of clinical practice. The effort to identify cutoff IFN values for degree of disease activity or remission are not helpful (could be eliminated from the manuscript) and the ROC curves shown in Supplementary Figure 1 indicate that in the absence of other measurements, quantitation of IFN-a levels will have limited utility as a biomarker in clinical practice. Nonetheless, the data do support IFN-a levels as highly relevant to lupus disease, and they would be useful to incorporate into clinical practice as one of many factors to be considered in patient assessment. As noted by the authors, the Simoa assay is currently rarely available to clinicians and even for investigators it is quite expensive. Perhaps the data presented here could encourage clinical testing facilities to make the Simoa assay available to clinicians.

In addition to the use of the Simoa assay, strengths of the study include the wide spectrum of patients studied, with more than half characterized as in DORIS remission, and the detailed autoantibody data provided. Notable is the very strong positive correlation between autoantibodies specific for RNA-associated particles and the significant negative correlation with certain anti-phospholipid (APL) antibodies. The latter observation has been previously reported, including in a recent publication from Karolinska (Rheumatology, 2025, 64, 1469–1475) describing IFN-a levels in pregnant SLE patients. The distinction between autoantibodies targeting RNA-associated proteins and those among APL specificities in relation to activation of the IFN pathway was noted years ago, and the current manuscript’s data suggest an opportunity for further investigation of the role of the antiphospholipid antibodies in regulating IFN expression.

The manuscript might be strengthened by tightening up the Introduction and Discussion. Much of the Introduction simply reviews background related to interferons and is probably unnecessary for most readers. The authors (in the first paragraph) note the role of cytoplasmic nucleic acid sensors in induction of IFN-a but they fail to describe the role of nucleic acid-containing immune complexes in inducing IFN-a through Toll-like receptors, likely the major mechanism. The role of TLRs should be recognized.

The Discussion is also excessively long and does not provide any major insights into the biologic significance of the findings. Shortening the manuscript to just present the data with a brief acknowledgement of other relevant publications would be helpful.
