# Supplementary material for: Ultrasensitive quantification of serum IFN-α and IFN-γ in systemic lupus erythematosus: A cross-sectional observational study
Source: PLoS Med. 2025 Dec 5;22(12):e1004841. doi: 10.1371/journal.pmed.1004841 (PMC12680241; doi:10.1371/journal.pmed.1004841)
Supplement: Attachment — Submitted filename: Response to Reviewers PMEDICINE-D-25-02814R1.docx [file pmed.1004841.s010.docx]

Editor-in-Chief,

PLOS Medicine

Tenerife, September 14, 2025

Dear Editor,

Thank you very much for giving us the opportunity to submit a revised version of our manuscript entitled “Ultrasensitive Quantification of Serum IFN-α, But Not IFN-γ, Reflects Inflammation, Disease Activity, and Autoantibody Status in Systemic Lupus Erythematosus”, PMEDICINE-D-25-02814R1.

Below please find our responses to the Reviewers' comments. You will also see that the responses have been incorporated into the revised manuscript using Word’s tracked changes.

Sincerely yours,

Professor Miguel González-Gay	Dr. Iván Ferraz-Amaro

Hospital Univ. Marqués de Valdecilla	Hospital Universitario de Canarias

Santander, Spain	Tenerife, Spain

**Reviewer #1**

**Gonzalez-Gay et al. present a very nice data set relating serum interferon-alpha and -gamma levels measured by high sensitivity Simoa assay to a number of demographic and clinical parameters in 313 patients with SLE. While the observations presented are not novel and generally confirm previously published studies, the availability of IFN levels across a large number of patients allows for a multivariate analysis of correlations that emphasize the significance of IFN-a (but not IFN-gamma) as a mediator with potential to assist in characterizing disease activity and possibly organ damage in the context of clinical practice. The effort to identify cutoff IFN values for degree of disease activity or remission are not helpful (could be eliminated from the manuscript) and the ROC curves shown in Supplementary Figure 1 indicate that in the absence of other measurements, quantitation of IFN-a levels will have limited utility as a biomarker in clinical practice. Nonetheless, the data do support IFN-a levels as highly relevant to lupus disease, and they would be useful to incorporate into clinical practice as one of many factors to be considered in patient assessment. As noted by the authors, the Simoa assay is currently rarely available to clinicians and even for investigators it is quite expensive. Perhaps the data presented here could encourage clinical testing facilities to make the Simoa assay available to clinicians.**

REPLY: We thank the Reviewer for their positive comments on our work.

The Reviewer is absolutely right, and in this regard, we acknowledge that our attempt to identify IFN cutoffs for discriminating disease activity was unsuccessful, as ROC curve analysis demonstrated poor discriminatory ability. While we understand that the IFN cutoff results may not provide substantial novel information and could be removed, we believe there is still value in presenting these data for completeness and transparency. Therefore, we have relocated Table 3 to the supplementary material (S1 Table) and removed the cutoff results from the summary. Instead, the summary now indicates, in more general terms, that these cutoffs provided limited diagnostic value.

**In addition to the use of the Simoa assay, strengths of the study include the wide spectrum of patients studied, with more than half characterized as in DORIS remission, and the detailed autoantibody data provided. Notable is the very strong positive correlation between autoantibodies specific for RNA-associated particles and the significant negative correlation with certain anti-phospholipid (APL) antibodies. The latter observation has been previously reported, including in a recent publication from Karolinska (Rheumatology, 2025, 64, 1469–1475) describing IFN-a levels in pregnant SLE patients. The distinction between autoantibodies targeting RNA-associated proteins and those among APL specificities in relation to activation of the IFN pathway was noted years ago, and the current manuscript’s data suggest an opportunity for further investigation of the role of the antiphospholipid antibodies in regulating IFN expression.**

REPLY: We again thank the Reviewer for his constructive comments, which clearly improve the quality of the manuscript. In this regard, we appreciate the reviewer's interesting suggestion regarding this recent publication, of which we were previously unaware, and which supports our findings. We have therefore added this citation and analyzed its results against ours on page 13, lines 469-472.

**The manuscript might be strengthened by tightening up the Introduction and Discussion. Much of the Introduction simply reviews background related to interferons and is probably unnecessary for most readers. The authors (in the first paragraph) note the role of cytoplasmic nucleic acid sensors in induction of IFN-a but they fail to describe the role of nucleic acid-containing immune complexes in inducing IFN-a through Toll-like receptors, likely the major mechanism. The role of TLRs should be recognized.**

**The Discussion is also excessively long and does not provide any major insights into the biologic significance of the findings. Shortening the manuscript to just present the data with a brief acknowledgement of other relevant publications would be helpful.**

REPLY: The Reviewer correctly points out that the text was long and that greater conciseness would improve its clarity and understanding. Consequently, the *Introduction* and *Discussion* sections have been shortened. In particular, the description of the physiological mechanisms of IFN has been condensed in the Introduction section. In addition, the role of TLRs in IFN pathways has been briefly described. (page 5, lines 123-127).

**Reviewer #2**

**I only reviewed the methods and statistics of this paper. There are several major concerns:**

**1. Confounders selected based on p-values are not appropriate for 2 reasons: p-values are not designed for variable selection and doing so could inflate type 1 errors; this practice does not account for the causality between variables - confounders by definition should be a variable causing exposure and outcome. If the author intends for a prediction (which has not been clear) they should consider either using AIC for variable selection or LASSO.**

REPLY: We appreciate the Reviewer's insightful comments on variable selection for confounders. We want to clarify that our study does not focus on predictive modeling, but rather on the study of associations, specifically to estimate the effect of an independent variable (disease characteristics) on a dependent outcome (serum interferon levels). In this context, the focus is on controlling confounding bias rather than optimizing predictive accuracy. Therefore, we selected confounders based on a criterion of a 10% change in the beta coefficient estimate, an approach widely used in epidemiological studies to identify variables that significantly influence the association between exposure and outcome. This approach aligns with our goal of obtaining an unbiased estimate of effect rather than building a predictive model. Consequently, the multivariate beta coefficients have been recalculated in Table 2 using this approach. Furthermore, the methodology of this multivariable analysis is described in the Statistical Analysis section. (page 8, lines 308-315).

**2. Relatedly, if that analysis aims for causal inference, it should be cautioned that cross-sectional design would mean reverse causation is possible.**

REPLY: We thank the Reviewer for their insightful comment. In this regard, while we acknowledged this limitation in the original manuscript, we now emphasize in the revised version that the cross-sectional study design limits causal inference and does not allow for definitively establishing the directionality of the associations, including the possibility of reverse causality (page 15, line 576-577)

**3. Log-transformation was done to account for non-normally distributed variables but have the authors examine whether the transformed variables are 'sufficiently normal'?**

REPLY: Yes, indeed. After log transformation, both IFN-α and IFN-γ were confirmed to follow a normal distribution. The method used to assess the normality of log-transformed variables has been included in the *Statistical Analysis* section (page 9, line 317-319) of the revised manuscript.

**4. It is clear how the 'optimal cut-off' is determined - is it by maximising the sum of sensitivity ana specificity?**

REPLY: We appreciate the opportunity to clarify this point. The optimal cutoff in our study was determined using the maximum efficiency method, which balances the costs of false negatives and false positives (cost of FN = cost of FP), and was adjusted considering the observed prevalence in the study population. This approach seeks to maximize the overall classification accuracy, rather than simply maximizing the sum of sensitivity and specificity. This has been clarified in the *Statistical Analysis* section (page 9, lines 323-326).

**5. The AUC and sensitivity shown are really low - I'm not sure whether this is clinically relevant / useful.**

REPLY: The Reviewer is absolutely correct. Both the AUC and sensitivity values were low in our analysis, which was a key finding of our work. For this reason, we acknowledged in the manuscript that the proposed serum interferon cutoff levels had low discriminatory power and lacked clinical relevance.

**6. (minor) would be good to show Table 1 by the outcome.**

REPLY: We thank the Reviewer for this suggestion. However, our study was not prospective, nor did it include a binary outcome measure in the usual sense of this type of study. Instead, it investigated the relationship between the clinical and demographic characteristics of SLE patients and their serum interferon levels. Therefore, we respectfully consider that it would not be appropriate to stratify patients by outcome in Table 1.

**Reviewer #3**

**I carefully read the article by Gonzalez-Gay et al. entitled "Ultra-sensitive quantification of serum IFN-α, but not IFN-γ, reflects inflammation, disease activity, and autoantibody status in systemic lupus erythematosus." This study demonstrates that serum IFN-α, measured with an ultra-sensitive immunoassay, is a valuable biomarker of SLE activity. It confirms findings from other groups in the field, using a large cohort of patients with this disease. The data also show that IFN-γ is a less reliable marker of SLE activity compared to IFN-α. As studies using ultra-sensitive immunoassays to quantify IFN-α in SLE remain scarce, this work is an important contribution to the scientific community. However, the clinical utility of ultra-sensitive assays appears less critical for IFN-γ than for IFN-α, given the higher circulating serum concentrations of the former. The manuscript is well-structured, concise, and easy to follow. However, several aspects require revision and, hopefully, improvement from the authors.**

**Major points**

***	For clinicians, perhaps the most striking—and disappointing—result is that serum IFN-α levels, although correlated with disease activity, do not reliably discriminate between clinically active and inactive patients, regardless of the activity scores used. This finding contrasts with previous reports (Mathian, Arthritis Rheumatol 2019, PMID: 30507062; Wahadat MJ, Rheumatology 2023, PMID: 36515466). It is surprising that the present study identifies discriminant thresholds as high as >10-15 pg/mL, whereas earlier studies reported much lower cut-offs (266 fg/mL in Mathian et al., 225.9 fg/mL in Wahadat et al.). Such high IFN-α levels are usually observed in clinically overtly active SLE and are rarely found in patients with minimal disease manifestations. These discrepancies likely reflect differences in assay technologies, pre-analytical processes, and possibly patient characteristics. Of note, the Gonzalez-Gay cohort included relatively few clinically active patients, with poorly detailed distribution of activity levels and no description of clinical involvement types. The authors acknowledge these limitations, including the inability to test their platform on healthy controls.**

**Furthermore, the use of SLEDAI and SLEDAS in this context raises concerns, as these scores include serological markers (complement and anti-dsDNA), which are not clinically meaningful indicators of clinical activity. For instance, the authors report 45% inactive versus 55% active patients. Yet, they also report 68% patients in remission. This implies that, at most, only 32% had clinical evidence of activity—likely fewer, since many of those not in remission were classified so merely due to prednisone >5 mg/day, despite clinical inactivity. It is probable that the 23% discrepancy (55%-32%=23%) represents patients with only serological abnormalities (complement consumption and/or elevated anti-dsDNA), which are of little clinical relevance.**

**To clarify these issues and assist readers in interpreting the data, I suggest the following:**

**o	Provide detailed graphical presentations of individual IFN-α values and medians across subgroups (by SLEDAI activity level: none/mild/moderate/high; by SLE-DAS activity levels; remission/mild/moderate/severe; remission vs non-remission; LLDAS vs non-LLDAS). This would allow readers to assess dispersion and subgroup sizes, as well as to visualize how many patients fall above or below ROC-derived thresholds.**

REPLY: We thank the Reviewer for his thoughtful comments on our manuscript. We also thank the Reviewer for this valuable suggestion. Because of that, a graphic representation of IFN-α levels according to the SLEDAI and SLEDAS categories, as well as between the non-DORIS and DORIS, and non-LLDAS and LLDAS subgroups, is included in Figure 2.

**o	For ROC analyses, repeat calculations excluding purely serological activity by using the clinical SLEDAI (i.e., omitting complement and anti-dsDNA components). Many clinically inactive patients are misclassified as active under standard SLEDAI ≥4, while some with overt manifestations fall below this cut-off. Similar considerations may apply to SLE-DAS if a "clinical" version exists. Additionally, for comparison, I strongly encourage ROC analyses of C3, C4, and anti-dsDNA against clinical SLEDAI, DORIS, and LLDAS.**

**o	Expand the discussion by comparing the proportion of active patients in this study with other published cohorts.**

REPLY: Again, we thank you for this interesting suggestion. We calculated the clinical SLEDAI-2K by excluding the complement and anti-DNA items. Accordingly, we performed a ROC curve analysis of IFN-α against this clinical SLEDAI-2K. This information has been incorporated into Table 3 (which is now S1 Table, as recommended by Reviewer #1) and S2 Figure. In addition, modified LLDAS and SLEDAI-2K >0 have also been included in the ROC analysis.

After this reanalysis, most scores still yielded cutoffs in thousands of femtograms, except for SLEDAI-2K >0, which resulted in a cutoff of 39 fg/ml, consistent with previous reports. However, in the study by Mathian et al. (PMID 30507062), the median SLEDAI was 4 (range: 0–36), with 55% of patients having a SLEDAI greater than 4, and in the study by Wahadat et al. (PMID 36515466), the median clinical SLEDAI was 3 (range: 0–14). This contrasts with our cohort, where the median SLEDAI was 2 (IQR: 0–4), the median clinical SLEDAI was 0 (IQR: 0–1), and only 26% of patients had a SLEDAI ≥4. In keeping with some of the comments raised by the Reviewer, we believe that these differences in disease activity between the study populations likely explain the discrepancies in the cutoff values identified compared with previous reports. We have discussed these aspects in more detail in the revised version of the manuscript to improve clarity and consistency (page 14, lines 523-529).

We are not aware of any ‘clinical’ SLE-DAS described so far in the literature. Regarding the exclusion of laboratory parameters from the SLEDAS calculation, from a clinical perspective, the calculation seems difficult to perform. In this regard, SLEDAS includes clinical and laboratory parameters, uses a complex formula, and, in clinical practice, requires the use of a calculator.

On the other hand, in our study, the variables anti-dsDNA, C3, and C4 were collected as binary measures: positivity for anti-dsDNA and presence or absence of hypocomplementemia, respectively. Although ROC curve analysis can be performed with binary independent variables, we believe such an analysis would lack sufficient power to determine an optimal cutoff point. We acknowledge the relevance of the analysis suggested by the Reviewer. However, it cannot be performed with our dataset in its current form. We understand that the Reviewer’s aim is to assess whether cutoff points for C3, C4, and anti-dsDNA levels could be established to discriminate among different scores, and whether these cutoffs would be comparable to those for IFN. We recognize that such an analysis is not feasible with the available data, and we consider that establishing a reliable comparison may be beyond the scope of our study.

We have compared the proportion of active patients in this study with other cohorts published on page 14 lines 523-529.

***	The discussion misrepresents the work of Mathian et al. (Mathian, Arthritis Rheumatol 2019, PMID: 30507062). The threshold of 266 fg/mL was derived using ROC curves with clinical activity (clinical SLEDAI >0) as the gold standard, not SLEDAI ≥4. This point requires correction.**

REPLY: We apologize for this mistake. The correction has been made on page 14, line 522.

***	Damage indices such as SDI integrate cumulative disease and treatment consequences over many years (mean disease duration ~19 years in this cohort). It is biologically implausible that a short-term biomarker such as serum IFN-α, which can fluctuate within minutes, reflects cumulative damage. Without longitudinal IFN-α data, such analyses should be avoided.**

REPLY: Based on the thoughtful comment raised by the Reviewer, the association of SLICC with IFN values has been removed from Tables 2 and S1 Table, as well as from S2 Figure. In addition, all references to SLICC findings have been removed from the *Abstract* and *Results* sections.

***	Additional analyses of modified LLDAS (≤5 mg/day prednisone instead of 7.5 mg) would be informative, given its status as a current treatment target (Fanouriakis A, Ann Rheum Dis 2024, PMID: 37827694).**

REPLY: We thank the Reviewer for this interesting suggestion. Due to this, the relationship between a modified LLDAS, defined using a prednisone dosage cutoff of ≤5 mg/day instead of the standard ≤7.5 mg/day, and IFN-α levels has been incorporated into the Table 2. In addition, calculation of modified LLDAS has been included in the *Methods* section using Fanouriakis et al. reference (page 8, line 277).

***	In Table 2, cytokine levels should be reported for each subgroup, including measures of dispersion, to fully inform the reader.**

REPLY: We are pleased to provide the Reviewer with further clarification on this matter. Specifically, we would like to explain that Table 2 presents the associations between disease-related variables and serum IFN-α and IFN-γ levels, modeling these cytokines as continuous dependent variables in regression analyses. In this context, the beta coefficients represent the difference in IFN values between groups for binary predictors, and the association with log-transformed IFN values for continuous predictors. As such, it is not feasible to report cytokine levels or measures of dispersion by subgroup within this table, as the analysis does not generate such data. The 95% CI, in fact, represents the measure of dispersion for the beta coefficient. We believe the current presentation most accurately reflects the relationships being studied. However, we appreciate the Reviewer’s suggestion and would be happy to provide additional subgroup summaries if deemed necessary.

***	Table 2 would be more clinically interpretable if results were expressed as odds ratios rather than beta coefficients.**

REPLY: We again thank the Reviewer for the exhaustive and helpful analysis of our study. In Table 2, IFN is modeled as a continuous variable rather than categorized into binary groups based on a specific cutoff value. Accordingly, we applied linear regression, which estimates associations using beta coefficients rather than odds ratios. To the best of our knowledge, odds ratios are appropriate for models with binary or categorical outcome variables (e.g., logistic regression), whereas beta coefficients describe the change in the continuous outcome per unit change in the predictor. For this reason, we respectfully believe that presenting the results as odds ratios in Table 2 would not be statistically appropriate or interpretable.

**Minor points**

***	Pre-analytical conditions must be detailed (sample type, storage, and handling), as IFN-α is labile and requires strict preservation protocols.**

REPLY: We confirm that all pre-analytical conditions were strictly controlled to ensure sample integrity. Serum samples were collected following standard protocols, immediately aliquoted, and frozen at -80ºC without delay. Samples were stored continuously at -80ºC until assay to preserve the labile nature of IFN-α. These procedures conform to recommended best practices to ensure the stability and reliability of IFN-α measurements. In response to the Reviewer’s comment, this information has been added to the *Methods* section on page 8, lines 282-285.

***	In Table 1, several percentages appear inconsistent. My recalculations yield: dyslipidemia 56% (vs 58%), metabolic syndrome 44% (vs 45%), SLEDAS remission 76% (vs 78%), anti-ENA 70% (vs 71%), anticardiolipin IgG 15% (vs 16%). These discrepancies should be corrected.**

REPLY: The discrepancies noted by the reviewer are due to missing data. To address this, Table 1 has been updated to indicate the amount of missing data for each variable, thereby improving transparency and avoiding such discrepancies.

***	The claim that this is the first multivariable analysis of IFN-α clinical associations is inaccurate; such analyses have been reported (Mathian, Arthritis Rheumatol 2019, PMID: 30507062).**

REPLY: This mistake has been corrected on page 10 lines 450-451.

***	The study does not reference Chasset et al. (Ann Rheum Dis 2022, PMID), one of the earliest to compare ultra-sensitive immunoassays for IFN-α, IFN-γ, and type I IFN scores in relation to SLE activity. This work should be discussed.**

REPLY: We thank the reviewer, as the inclusion of this study undoubtedly improves the quality of the discussion. Therefore, this important study has been included in the text and is discussed on page 13, lines 469-472.

**General editorial requests:**

**(Note: not all will apply to your paper, but please check each item carefully)**

***	We ask every co-author listed on the manuscript to fill in a contributing author statement, making sure to declare all competing interests. If any of the co-authors have not filled in the statement, we will remind them to do so when the paper is revised. If all statements are not completed in a timely fashion this could hold up the re-review process. If new competing interests are declared later in the revision process, this may also hold up the submission. Should there be a problem getting one of your co-authors to fill in a statement we will be in contact. Please do not add or remove authors without first discussing this with the handling editor. You can see our competing interests policy here: http://journals.plos.org/plosmedicine/s/competing-interests.**

REPLY: A file containing Competing Interests' statement of all the authors has been submitted. We have not found any specific form or document to fill out on the website. If the attached document is not sufficient, we would be happy to provide any additional information or document required.

***	Please upload any figures associated with your paper as individual TIF or EPS files with 300dpi resolution at resubmission; please read our figure guidelines for more information on our requirements: http://journals.plos.org/plosmedicine/s/figures. While revising your submission, we strongly recommend that you use PLOS's NAAS tool (https://ngplosjournals.pagemajik.ai/artanalysis) to test your figure files. NAAS can convert your figure files to the TIFF file type and meet basic requirements (such as print size, resolution), or provide you with a report on issues that do not meet our requirements and that NAAS cannot fix.**

**After uploading your figures to PLOS's NAAS tool - https://ngplosjournals.pagemajik.ai/artanalysis, NAAS will process the files provided and display the results in the "Uploaded Files" section of the page as the processing is complete.**

**If the uploaded figures meet our requirements (or NAAS is able to fix the files to meet our requirements), the figure will be marked as "fixed" above. If NAAS is unable to fix the files, a red "failed" label will appear above.**

**When NAAS has confirmed that the figure files meet our requirements, please download the file via the download option, and include these NAAS processed figure files when submitting your revised manuscript.**

REPLY: We have tested all figures from our manuscript using the PLOS tool, and for each one we received the message: “Image is adjusted to match the specification”. The figures after downloaded have been submitted with our revised manuscript.

***	Please ensure that the paper adheres to the PLOS Data Availability Policy (see http://journals.plos.org/plosmedicine/s/data-availability), which requires that all data underlying the study's findings be provided in a repository or as Supporting Information. For data residing with a third party, authors are required to provide instructions with contact information (web or email address) for obtaining the data. Please note that a study author cannot be the contact person for the data. PLOS journals do not allow statements supported by "data not shown" or "unpublished results." For such statements, authors must provide supporting data or cite public sources that include it.**

REPLY: Data underlying the study's findings is provided in a repository. We have included the statement in manuscript’s page 16 as “The dataset supporting the findings of this study is openly available at Zenodo: <https://zenodo.org/records/17106069>”.

***	We expect all researchers with submissions to PLOS in which author-generated code underpins the findings in the manuscript to make all author-generated code available without restrictions upon publication of the work. In cases where code is central to the manuscript, we may require the code to be made available as a condition of publication. Authors are responsible for ensuring that the code is reusable and well documented. Please make any custom code available, either as part of your data deposition or as a supplementary file. Please add a sentence to your data availability statement regarding any code used in the study, e.g. "The code used in the analysis is available from Github [URL] and archived in Zenodo [DOI link]" Please review our guidelines at https://journals.plos.org/plosmedicine/s/materials-software-and-code-sharing and ensure that your code is shared in a way that follows best practice and facilitates reproducibility and reuse. Because Github depositions can be readily changed or deleted, we encourage you to make a permanent DOI'd copy (e.g. in Zenodo) and provide the URL.**

REPLY: We confirm that no author-generated or custom code was used in the analyses or reported findings of this study.

***	Thank you for agreeing to make your data available. At this time, please provide the link to the data repository and accession numbers required for access.**

REPLY: https://zenodo.org/records/17106069

***	The funding statement should include: specific grant numbers, initials of authors who received each award, URLs to sponsors’ websites. Also, please state whether any sponsors or funders (other than the named authors) played any role in study design, data collection and analysis, the decision to publish, or preparation of the manuscript. If they had no role in the research, include this sentence: “The funders had no role in study design, data collection and analysis, decision to publish, or preparation of the manuscript.”**

REPLY: This information has been fully included in page 16 and as a Supporting Information file.

**FORMATTING - GENERAL**

***	Abstract: Please structure your abstract using the PLOS Medicine headings (Background, Methods and Findings, Conclusions). Please combine the Methods and Findings sections into one section.**

REPLY: This has been modified in page 3.

***	At this stage, we ask that you include a short, non-technical Author Summary of your research to make findings accessible to a wide audience that includes both scientists and non-scientists. The Author Summary should immediately follow the Abstract in your revised manuscript. This text is subject to editorial change and should be distinct from the scientific abstract. Ideally each sub-heading should contain 2-3 single sentence, concise bullet points containing the most salient points from your study. In the final bullet point of 'What Do These Findings Mean?', please include the main limitations of the study in non-technical language. Please see our author guidelines for more information: https://journals.plos.org/plosmedicine/s/revising-your-manuscript#loc-author-summary.**

REPLY: Authors summary has been included in the manuscript (page 4).

***	Please express the main results with 95% CIs as well as p values. When reporting p values please report as p<0.001 and where higher as the exact p value p=0.002, for example. Throughout, suggest reporting statistical information as follows to improve clarity for the reader "22% (95% CI [13%,28%]; p</=)". Please be sure to define all numerical values at first use.**

REPLY: This has been corrected.

***	Please include page numbers and line numbers in the manuscript file. Use continuous line numbers (do not restart the numbering on each page).**

REPLY: Page numbers and line numbers are included throughout the test.

***	Please cite the reference numbers in square brackets. Citations should precede punctuation.**

REPLY: This information was already correct in the manuscript and required no changes.

**FIGURES AND TABLES**

***	Please provide titles and legends for all figures and tables (including those in Supporting Information files).**

REPLY: A new file with titles and legends for all figures and tables has been submitted as a Supporting Information file.

***	Please define all abbreviations used in each figure/table (including those in Supporting Information files).**

REPLY: Abbreviations used in each figure/table are included in the Supporting Information file that includes titles and legends for all figures and tables.

***	Please consider avoiding the use of red and green in order to make your figure more accessible to those with color blindness.**

REPLY: Figure colors have been converted to grayscale.

**SUPPLEMENTARY MATERIAL**

***	Please note that supplementary material will be posted as supplied by the authors. Therefore, please amend it according to the relevant comments outlined here.**

REPLY: Supplementary material has been edited accordingly.

***	Please cite your Supporting Information as outlined here: https://journals.plos.org/plosmedicine/s/supporting-information**

REPLY: Format has been applied to supporting information.

**REFERENCES**

***	PLOS uses the numbered citation (citation-sequence) method and first six authors, et al.**

REPLY: Citations format has been updated.

***	Please ensure that journal name abbreviations match those found in the National Center for Biotechnology Information (NCBI) databases (http://www.ncbi.nlm.nih.gov/nlmcatalog/journals), and are appropriately formatted and capitalised.**

REPLY: Citations format has been updated.

***	Where website addresses are cited, please include the complete URL and specify the date of access (e.g. [accessed: 12/06/2023]).**

REPLY: URL do not apply in our manuscript. However, date of access has been added to Funding section URLs.

***	Please also see https://journals.plos.org/plosmedicine/s/submission-guidelines#loc-references for further details on reference formatting.**

REPLY: Citations format has been updated according to the journal style.

**STUDY TYPE-SPECIFIC REQUESTS**

***	In the manuscript text, please indicate: (1) the specific hypotheses you intended to test, (2) the analytical methods by which you planned to test them, (3) the analyses you actually performed, and (4) when reported analyses differ from those that were planned, transparent explanations for differences that affect the reliability of the study's results. If a reported analysis was performed based on an interesting but unanticipated pattern in the data, please be clear that the analysis was data driven.**

REPLY: We believe that all these requests have already been addressed in the manuscript. For example, we did not anticipate that interferon levels would deviate from a normal distribution or that the ROC curve results would be weak. Although this does not represent a data-driven analysis, these findings have been explained and discussed within the manuscript.

***	Please state in the Methods section whether the study had a prospective protocol or analysis plan. If a prospective analysis plan (from your funding proposal, IRB or other ethics committee submission, study protocol, or other planning document written before analyzing the data) was used in designing the study, please include the relevant document(s) with your revised manuscript as a Supporting Information file to be published alongside your study and cite it in the Methods section. A legend for this file should be included at the end of your manuscript. If no such document exists, please make sure that the Methods section transparently describes when analyses were planned, and when/why any data-driven changes to analyses took place. Changes in the analysis, including those made in response to peer review comments, should be identified as such in the Methods section of the paper, with rationale.**

REPLY: No prospective statistical analysis plan was registered prior to data collection. The analyses reported were pre-specified as part of the study design and detailed in the study protocol approved by the Institutional Review Board. Deviations from the initial plan were limited to the exclusion of ROC analyses for IFN-γ, which were not pursued since this cytokine did not show relevant associations with clinical or laboratory measures (page 6, lines 203-206). These deviations are not related to study design but the has been transparently described and discussed within the manuscript.

***	Please ensure that the study is reported according to the STARD guideline (https://www.equator-network.org/reporting-guidelines/stard/) and include the completed STARD checklist as Supporting Information. Please add the following statement, or similar, to the Methods: "This study is reported as per the Standards for Reporting of Diagnostic Accuracy (STARD) guideline (S1 Checklist)." When completing the checklist, please use section and paragraph numbers, rather than page numbers.**

***	Please structure your Abstract according to STARD for Abstracts (https://www.equator-network.org/reporting-guidelines/stard-abstracts/).**

REPLY: The STARD (Standards for Reporting of Diagnostic Accuracy Studies) guidelines are specifically intended for studies evaluating the diagnostic accuracy of medical tests, which was not the focus of our work. Therefore, we believe adherence to STROBE—and not STARD—is appropriate for this manuscript. Accordingly, The STROBE (Strengthening the Reporting of Observational Studies in Epidemiology) guidelines were followed for this study, as our research employed a cross-sectional observational design to assess associations between biomarker levels and clinical features. We have included “This study is reported as per the STROBE (Strengthening the Reporting of Observational Studies in Epidemiology) guideline (S1 Checklist)” in page 6 lines 202.

***	Please structure the Methods section using the following sub-headings: Study design, Participants, Test methods, Analysis.**

REPLY: Methods has been structured in Study design, Study participants, Data collection, Laboratory measurements and Statistical analysis.

***	Please include a diagram to describe the flow of participants through the study (typically figure 1).**

REPLY: A flowchart depicting the selection process of the patients has been included as Supplementary Figure 1 (S1 Figure).

***	Address the extent to which the study population is representative of the population of interest.**

REPLY: This has been clarified in page 15, lines 569-575.
