# Supplementary material for: Ultrasensitive quantification of serum IFN-α and IFN-γ in systemic lupus erythematosus: A cross-sectional observational study
Source: PLoS Med. 2025 Dec 5;22(12):e1004841. doi: 10.1371/journal.pmed.1004841 (PMC12680241; doi:10.1371/journal.pmed.1004841)
Supplement: Attachment — Submitted filename: Response to Reviewers PMEDICINE-D-25-02814R2.docx [file pmed.1004841.s011.docx]

Editor-in-Chief,

*PLOS Medicine*

Tenerife, November 11, 2025

Dear Editor,

Thank you very much for giving us the opportunity to submit a revised version of our manuscript entitled “***Ultrasensitive Quantification of Serum IFN-α, But Not IFN-γ, Reflects Inflammation, Disease Activity, and Autoantibody Status in Systemic Lupus Erythematosus***”, PMEDICINE-D-25-02814R2.

Below please find our responses to the Reviewers' comments. You will also see that the responses have been incorporated into the revised manuscript using Word’s tracked changes.

Sincerely yours,

Professor Miguel González-Gay	Dr. Iván Ferraz-Amaro

Hospital Univ. Marqués de Valdecilla	Hospital Universitario de Canarias

Santander, Spain	Tenerife, Spain

**Comments from Reviewers:**

**Reviewer #1: The authors have made a good effort to respond to all reviewer comments. While the study has limitations - including the high number of patients in remission (although that is also a strength as it demonstrates that IFN-a is present in inactive as well as active patients) and the absence of information on the relationship of IFN-a to clinical features of SLE (such as organ involvement), it provides a nice dataset for consideration by other investigators. It also supports the utility of the Simoa platform for detection of IFN-a. The manuscript strongly makes the case for an association of autoantibodies targeting RNA-associated proteins with serum IFN-a and a negative association of anti-phospholipid antibodies with IFN-a, with the latter an association (negative) that cries out for more investigation.**

REPLY: We thank the Reviewer for their positive comments. The limitations commented by the Reviewer have been incorporated in the revised manuscript, specifically in lines 510-514.

**Reviewer #2: Thanks for addressing my comments and provide clarification to the methods and interpretation. One thing that I do not agree on is the use of 'data-driven' in selecting confounders. Variables that influence the exposure-outcome associations could be confounders (which should be adjusted) as well as mediators and colliders (should not be adjusted). This method does not tell us anyone on these. The authors should at least acknowledge that the method they used to induce overadjustment and collider bias.**

**Schisterman EF, Cole SR, Platt RW. Overadjustment bias and unnecessary adjustment in epidemiologic studies. Epidemiology. 2009 Jul 1;20(4):488-95.**

**Holmberg MJ, Andersen LW. Collider bias. Jama. 2022 Apr 5;327(13):1282-3.**

REPLY: The Reviewer is completely right. In our study, confounder selection was primarily theory‑ and design‑informed. We specified a causal framework based on prior literature and clinical knowledge to define a minimal sufficient adjustment set, and we used data‑driven procedures only. We appreciate this important point and agree that purely data‑driven variable selection cannot distinguish confounders from mediators or colliders and thus can risk overadjustment and collider bias. However, the Reviewer should consider that the use of mediation and collider bias analyses to select confounders is not commonly performed or reported in typical medical literature, especially observational studies in clinical research. While it is true that adjusting for mediators or colliders can induce bias, we believe acknowledging and discussing this limitation is more realistic than performing formal mediation or collider analyses, which are still relatively uncommon in the field. Accordingly, we have explicitly acknowledged the limitations the Reviewer notes and the potential for overadjustment/collider bias in the Abstract and in lines 519-522.

**Reviewer #3: I have carefully read the authors' responses to the questions raised during the first submission, as well as re-reviewed the revised manuscript in its entirety. The authors have satisfactorily addressed the concerns raised and modified the manuscript accordingly. 
I believe that two very minor adjustments would further improve the quality of the paper:**

***    In the new Figure 2: please add the sample size for each subgroup (n = X). In the legend, indicate explicitly that this is a Box-and-Whisker plot, thus showing medians, quartiles, and extremes. It would also be helpful to include on the figure the thresholds derived from the ROC curve AUC values.**

REPLY: Sample size has been added to each subgroup in Figure 2. Besides, boxplot has been described in the figure legend. Thresholds were included in S2 Figure. We understand showing thresholds apply to S2 Figure but not to Figure 2.

***    Page 13, line 360: unless I am mistaken, the authors state that "the cutoff values for remission according to DORIS and LLDAS were 10,800 and …". However, in the corresponding table the value of 11,400 is reported. Could the authors clarify this apparent discrepancy?**

REPLY: We thank the Reviewer for detecting these discrepancies. These mistakes have been corrected (line 366).

**------------------------------------------------------------
Requests from Editors:

GENERAL

* Please confirm that your title complies with to PLOS Medicine's style. Your title must be nondeclarative and not a question. It should begin with main concept if possible. "Effect of" should be used only if causality can be inferred, i.e., for an RCT. Please place the study design ("A randomized controlled trial," "A retrospective study," "A modelling study," etc.) in the subtitle (ie, after a colon).**

REPLY: Title has been changed to “Ultrasensitive Quantification of Serum IFN-α and IFN-γ in Systemic Lupus Erythematosus: A Cross-Sectional Observational Study”.

*** Statistical reporting: Please revise throughout the manuscript, including tables and figures.
- Please report statistical information as follows to improve clarity for the reader ""22% (95% CI [13,28]; p</=)"".
- Please separate upper and lower bounds with commas instead of hyphens as the latter can be confused with reporting of negative values.
- Please repeat statistical definitions (HR, CI etc.) for each set of parentheses.**

REPLY: A space has been maintained between the confidence interval bounds to improve readability and avoid overcrowding in the tables. If this is incorrect, please let me know, and I will make the necessary adjustments.

*** Thank you for agreeing to make your data available. Please ensure that the Data Availability Statement in the online submission form is updated.**

REPLY: The data set has not been modified during this second revision round. Zenodo link reflects the correct dataset.

*** Please ensure that all abbreviations are defined at first use throughout the text (including statistical abbreviations).**

REPLY: All abbreviations are defined at first use throughout the text.

*** Please ensure that tables and figures, including those in supplementary files, are appropriately referenced in the main text.**

REPLY: All the tables and figures of the manuscript are correctly referenced in the main text.

*** Please review your text for claims of novelty or primacy (e.g. 'for the first time' or ‘novel’) and remove this language.**

REPLY: We revised the manuscript to avoid claims of primacy or superlatives (e.g., ‘first,’ ‘largest,’ ‘most comprehensive’) and now describe the cohort and methods using neutral, factual language focused on sample size, phenotyping depth, and associations.

*** Please confirm that any use of statistical terms (such as trend or significant) are supported by the data, and if not please remove them. The term trend should be used only when the test for trend has been conducted.**

REPLY: ‘Trend’ is not used in our manuscript. When the word ‘significant’ is used, this means the statistical significance has been reached.

*** Please provide titles and legends for all figures and tables (including those in Supporting Information files).**

REPLY: Titles and legends of figures and tables are provided in a supplementary document submitted with the manuscript.

*** Please define all acronyms used in each figure or table in its corresponding legend.**

REPLY: Acronyms have been checked in each figure and table.

*** Please revise for use of patient-centered language. Please note that patient-centered language is constructed with the use of post-modified nouns (e.g. 'patients with psoriasis’ (or similar) instead of ‘psoriasis patients’) putting the person first in the sentence structure.**

REPLY: ‘Patients with SLE’ has been used throughout the text.

*** Your study is observational and therefore causality cannot be inferred. Please confirm that you have not used language that implies causality, but rather referred to associations.**

REPLY: I confirm in this revised version no causality implication is mentioned.

*** Please upload the study protocol as a "Supporting Information" file and upload an English version.**

REPLY: English version of study protocol has been submitted.

*** Please confirm that the metadata in the online submission form is updated and accurate.**

REPLY: We confirm that all metadata submitted in the online submission form have been carefully reviewed and are accurate and up to date, reflecting the information contained in the manuscript **ABSTRACT

* Please confirm that your abstract complies with our requirements, including providing all the information relevant to this study type** [**https://journals.plos.org/plosmedicine/s/submission-guidelines#loc-abstract**](https://journals.plos.org/plosmedicine/s/submission-guidelines#loc-abstract)

REPLY: Abstract has been modified to comply with the journal requirements.

*** Please confirm that all numbers presented in the abstract are present and identical to numbers presented in the main manuscript text.**

REPLY: This has been checked.

*** In the abstract, please include the important dependent variables that are adjusted for in the analyses.**

REPLY: Dependent variable has been clearly defined in the abstract.

*** Please include the study design, population (with baseline characteristics) and setting, number of participants, years during which the study took place, length of follow up, and main outcome measures.**

REPLY: This has been included in the abstract.

*** Please quantify the main results (with 95% CIs and p values).**

REPLY: This has been included in the abstract.

*** In the last sentence of the Abstract Methods and Findings section, please describe the main limitation(s) of the study's methodology.**

REPLY: Main limitations have been included now in the Methods and Findings section of the Abstract.

*** l.62, “demographic characteristics and traditional cardiovascular risk factors” – please specify.**

REPLY: Exact demographic characteristics and traditional cardiovascular risk factors have been specified.

*** Abstract Conclusions:
- Please address the study implications without overreaching what can be concluded from the data; the phrase "In this study, we observed ..." may be useful.**

REPLY: The phrase 'In this study, we observed ...' has been included to moderate the tone of the conclusion.

**- Please interpret the study based on the results presented in the abstract, emphasizing what is new without overstating your conclusions.**

REPLY: The interpretation of the study based on the results presented in the abstract has been reviewed, emphasizing the novel aspects without overstating the conclusions. **METHODS AND RESULTS 

* Please confirm that the ethical approval number provided covers ethical approval from all four participating hospitals.**

REPLY: Ethical approval number of each hospital has now been included. Hospital Universitario Doctor Negrín and Hospital Insular de Gran Canaria share the same number.

*** Thank you for providing your STROBE checklist. Please replace the page numbers with paragraph numbers per section (e.g. "Methods, paragraph 1"), since the page numbers of the final published paper may be different from the page numbers in the current manuscript.**

REPLY: A revised STROBE checklist has been submitted in accordance with the recommendations.

*** Table 1/2: Please change ‘Female, n (%)’ to ‘Sex, female, n (%)’.**

REPLY: This has been modified.

*** l.268ff, “While most SLE patients had no activity (45%), mild or moderate-high activity was present in respectively 46% and 9% of the patients as indicated by the SLEDAI score.” – It seems that most patients had mild or moderate-high activity.**

REPLY: This sentence has been rephrased.

*** l.272, “The SDI was 0 (IQR 0-1) and an SDI score of 1 or higher was found in 46% of patients.” – We assume this refers to "SLICC-DI" in Table 1. We recommend using the same abbreviation consistently throughout.**

REPLY: SDI has been replaced for SLICC-DI.

*** Figure 1: Please note that in the graph the p-value is 0.000. Also, would it be useful to align the y axis for the two distribution graphs to facilitate comparison?**

REPLY: Figure 1 has been revised in accordance with the suggestions.

*** l.292ff: Please clarify that you are discussing the results of the multivariable adjustment.**

REPLY: This has been emphasized in lines 323-332.

*** l.293ff: Please ensure to present the results with sufficient detail, e.g. SLEDAI-2K category ‘mild’ was not significantly associated with elevated IFN-α levels.**

REPLY: This has been corrected in lines 324-330.

*** S1 Table: For SLEDAS, it says ‘2.08’ while in the main text you write ‘2.8’ (l.316). Please check.**

REPLY: This mistake has been corrected (line 359).

*** l.321, “while levels above 29,000 femtograms/ml discriminated high disease activity” – Should the reader be able to find this number in Table S1? Please carefully check the numbers reported in the paragraph (vs. the table).**

*** l.322, “The cutoff values for remission according to DORIS and LLDAS were 10,800…” – according to S1 Table the cutoff value for DORIS is 11,400.**

REPLY: These mistakes have been corrected (line 364).

*** Figure 1: Please include the n-numbers for each graph. Are the underlying numbers available in the Zenodo files?**

REPLY: N-numbers for each graph have been included in Figure 1. Yes, underlying numbers are available in the Zenodo file.

*** Figure 2: Please define all elements of box plots in the figure caption - center line, box limits and whiskers. Please include the n-number. Please specify ‘no activity’ (‘no disease activity’). Given a broad medical audience, we recommend that you provide a more detailed definition of "DORIS remission" and "LLDAS remission."**

REPLY: Boxplots elements have been defined in Figure 2 legend. N-number of subgroups have been added. DORIS and LLDAS definitions have been expanded.

*** Please provide the unadjusted comparisons as well as the adjusted comparisons in all relevant Tables (or as supplementary analysis).**

REPLY: Figures present unadjusted data. Tables 2 include both unadjusted and adjusted analyses. Multivariable analyses were not performed for Table 1 and Supplementary Table 1.

*** Please confirm that you have specified the variables controlled for in all relevant Tables.**

REPLY: We have added detailed definitions of the confounders considered to Table 2 to enhance clarity and assist readers in understanding the adjusted analyses.

**DISCUSSION

* When revising the discussion, please aim to streamline it and keep in mind that the journal is intended for a broad medical audience.**

REPLY: We believe that the discussion has been written with a broad medical audience in mind. Efforts were made to present the findings clearly and accessibly, avoiding overly technical jargon while maintaining scientific rigor.

*** l.361, “In this regard, our study represents the largest investigation to date…” – please add, ‘to our knowledge’.**

REPLY: This has been corrected in line 416.

*** l.459, “In conclusion, elevated serum levels of IFN-α identify patients with SLE exhibiting higher
460 disease activity, alongside increased ANA expression.” – please revise to focus on the associational aspect of the study.**

REPLY: This sentence has been revised accordingly (lines 523-524). **------------------------------------------------------------
General Editorial Requests

1) We ask every co-author listed on the manuscript to fill in a contributing author statement. If any of the co-authors have not filled in the statement, we will remind them to do so when the paper is revised. If all statements are not completed in a timely fashion this could hold up the re-review process. Should there be a problem getting one of your co-authors to fill in a statement we will be in contact. YOU MUST NOT ADD OR REMOVE AUTHORS UNLESS YOU HAVE ALERTED THE EDITOR HANDLING THE MANUSCRIPT TO THE CHANGE AND THEY SPECIFICALLY HAVE AGREED TO IT.**

REPLY: Authors have not been modified and we will be please to fill in a contributing author statement for each of them.

**2) Please ensure that the paper adheres to the PLOS Data Availability Policy (see** [**http://journals.plos.org/plosmedicine/s/data-availability**](http://journals.plos.org/plosmedicine/s/data-availability)**), which requires that all data underlying the study's findings be provided in a repository or as Supporting Information. For data residing with a third party, authors are required to provide instructions with contact information for obtaining the data. PLOS journals do not allow statements supported by "data not shown" or "unpublished results." For such statements, authors must provide supporting data or cite public sources that include it.**

REPLY: All data underlying the study's findings is currently transparently provided in the Zenodo link https://zenodo.org/records/17106069.

**3) Please review your reference list to ensure that it is complete and correct. If you have cited papers that have been retracted, please include the rationale for doing so in the manuscript text, or remove these references and replace them with relevant current references. Any changes to the reference list should be mentioned in the rebuttal letter that accompanies your revised manuscript.**

REPLY: The reference list has been carefully reviewed, and to the best of our knowledge, it does not contain any retracted articles.
